# Hypoxia in Obesity and Diabetes: Potential Therapeutic Effects of Hyperoxia and Nitrate

**DOI:** 10.1155/2017/5350267

**Published:** 2017-05-08

**Authors:** Reza Norouzirad, Pedro González-Muniesa, Asghar Ghasemi

**Affiliations:** ^1^Endocrine Physiology Research Center, Research Institute for Endocrine Sciences, Shahid Beheshti University of Medical Sciences, Tehran, Iran; ^2^Dezful University of Medical Sciences, Dezful, Iran; ^3^Centre for Nutrition Research, School of Pharmacy and Nutrition, University of Navarra, Pamplona, Spain; ^4^Department of Nutrition, Food Sciences and Physiology, School of Pharmacy and Nutrition, University of Navarra, Pamplona, Spain; ^5^IDISNA Navarra's Health Research Institute, Pamplona, Spain; ^6^CIBER Fisiopatología de la Obesidad y Nutrición (CIBERobn), Instituto de Salud Carlos III, Madrid, Spain

## Abstract

The prevalence of obesity and diabetes is increasing worldwide. Obesity and diabetes are associated with oxidative stress, inflammation, endothelial dysfunction, insulin resistance, and glucose intolerance. Obesity, a chronic hypoxic state that is associated with decreased nitric oxide (NO) bioavailability, is one of the main causes of type 2 diabetes. The hypoxia-inducible factor-1*α* (HIF-1*α*) is involved in the regulation of several genes of the metabolic pathways including proinflammatory adipokines, endothelial NO synthase (eNOS), and insulin signaling components. It seems that adipose tissue hypoxia and NO-dependent vascular and cellular dysfunctions are responsible for other consequences linked to obesity-related disorders. Although hyperoxia could reverse hypoxic-related disorders, it increases the production of reactive oxygen species (ROS) and decreases the production of NO. Nitrate can restore NO depletion and has antioxidant properties, and recent data support the beneficial effects of nitrate therapy in obesity and diabetes. Although it seems reasonable to combine hyperoxia and nitrate treatments for managing obesity/diabetes, the combined effects have not been investigated yet. This review discusses some aspects of tissue oxygenation and the potential effects of hyperoxia and nitrate interventions on obesity/diabetes management. It can be proposed that concomitant use of hyperoxia and nitrate is justified for managing obesity and diabetes.

## 1. Introduction

Obesity and diabetes, two major health problems worldwide, have shown an increasing trend in their prevalence over time [1]. Obesity, one of the main causes of type 2 diabetes [2], is associated with an increased number and size of triglyceride-filled white adipocytes [3]. Obesity is a state of hypoxia and low blood flow [4]. In this sense, hyperoxia exposure has been evaluated as a treatment for obesity and its related disorders [5, 6]. Although hyperoxia has shown beneficial effects for obesity management, it may result in nitric oxide (NO) depletion and induction of oxidative stress [7–9]. Interestingly, nitrate, a new proposed therapeutic agent for type 2 diabetes [10], restores NO depletion and has antioxidant properties [11]. This study reviews the effects of hyperoxia and nitrate interventions on the management of obesity and type 2 diabetes.

## 2. Adipose Tissue

There are three types of adipocytes or adipose tissues [12]. White adipose tissue (WAT) stores energy and lipids, in the form of triglycerides [3, 12]. Brown adipose tissue (BAT) dissipates energy and acts as a heat producer; BAT cells produce uncoupling protein 1 (UCP1), which uncouples the respiratory chain, that is, proton transport without phosphorylation, inducing thermogenesis instead of ATP production [13]. The third type is the brite or beige adipocytes [12]. Beige cells share some characteristics with BAT cells and others with WAT cells [14]. cAMP-induced UCP1 expression is higher in beige than in BAT cells; in addition, long-term treatment with thiazolidinedione (TZD) can induce higher UCP1 expression in beige cells than in WAT cells (8- to 10-fold versus 4- to 5-fold) [15].

Anatomically, adipose tissues are distributed in central adipose tissues (visceral and upper abdominal subcutaneous fats) and peripheral adipose tissues (hip and gluteofemoral fats) [16]. Visceral fat accumulation is associated with oxidative stress and inflammation [17, 18]. Adipose tissue is heterogeneous and includes adipocytes, vascular cells, and immune cells [16]. This tissue is metabolically active; for example, WAT is an endocrine organ and secretes a large variety of adipokines [19].  Table 1 shows some characteristics of different adipocytes.

## 3. Inflammation of Obese Adipose Tissue and Insulin Resistance

### 3.1. Obese Adipose Tissue

#### 3.1.1. Adipocyte Death and Turnover

In humans, the turnover of adipocytes is low, that is, ~10% per year, a rate that does not change in the early stages of obesity [22]. During the generation of WAT adipocytes, the expression of antiapoptotic factors such as B-cell lymphoma 2 (Bcl_2_) and flice-inhibitory protein (FLIP) leads to prolonged cell life [23]; insulin-like growth factor I (IGF-I) can decrease apoptosis of human fat cells by sustaining the antiapoptotic factors [24]. Bcl_2_ expression is negatively correlated with body mass index and inflammatory cytokines (interleukin-6 (IL-6) and tumor necrosis factor alpha (TNF-*α*)) [25]. Strissel et al. have reported that adipocyte death in epididymal adipose tissue of C57BL/6 male mice on a high-fat diet is increased from 0.1% at week 1 to 16% at week 12 [26]. In addition, the expression of proapoptotic caspases (CASP3, CASP7, CASP8, and CASP9) is increased, while the expression of Bcl_2_ (an antiapoptotic factor) is decreased in obesity [25]. Inflammatory factors such as TNF-*α*, IL-6, IL-1*β*, monocyte chemoattractant protein-1 (MCP1), and macrophage recruitment are also increased in obesity; these factors negatively affect adipocyte metabolism and its lipid storage capacity [2, 25, 27]. Rammos et al. have shown that 4 weeks of dietary nitrate (sodium nitrate 150 *μ*mol/kg body weight) administration can reduce macrophage migration inflammatory factor (MIF), which is a proinflammatory and atherogenic factor [28]. In obesity, BAT cell apoptosis is increased due to the decrease in Bcl_2_ [29] and increase in TNF-*α* [30]; however, low temperature can upregulate the Bcl_2_ gene expression and may protect BAT against apoptosis in cold situations [29].

#### 3.1.2. Lipolysis and Inflammation in Adipocytes

The functions of adipose tissues vary greatly between obese and lean subjects [25, 31]. For example, basal lipolysis is higher in obese adipose tissues than in lean ones [31]. In addition, elevated levels of fatty acids due to the increased lipolysis, high-fat diets, and hypoxia can result in ectopic fat deposition (as triglycerides and long-chain fatty acid forms) in skeletal muscles, liver, and *β*-cells; this increased ectopic fat deposition interferes with the normal functions of these tissues; for instance, the high levels of blood fatty acids and TNF-*α* occurring in obesity can induce insulin resistance [32–34]. Furthermore, long-term effects of medium- and long-chain fatty acids on *β*-cells are K^+^ channels opening and decrease in insulin secretion [35]. By contrast, fatty acids released due to lipolysis in lean subjects bind to coenzyme A to form acyl-CoA, which enters the *β*-oxidation pathway instead of the circulation, hence reducing the pernicious effects of an excessive free fatty acid release to the blood [2]. In inflammatory states, TNF-*α*, which is induced due to the increase in the numbers of both adipocytes and macrophages, inhibits normal differentiation of preadipocytes and induces proinflammatory phenotypes [36]. TNF-*α* inhibits peroxisome proliferator-activated receptor-*γ* (PPAR*γ*), which is involved in liver diseases and also lipid metabolism [37, 38]. Inhibition of PPAR*γ* increases circulating free fatty acids and therefore intensifies ectopic fat deposition in the liver, skeletal muscles, and other metabolic organs [2, 37, 38]. These conditions along with insulin resistance, low insulin production, and/or hyperphagia can worsen the situation and lead to hyperglycemia, glucose intolerance, and eventually diabetes.

#### 3.1.3. Adipose Tissue Macrophages and Inflammation

It has been estimated that the amount of adipose tissue macrophage infiltration in lean mice and humans is under 10% [39]. However, this amount is increased up to 50% in extremely obese mice and up to 40% in obese humans [39]. Recently, two types of macrophages have been described, based on their activation: M1, classically activated and M2, alternatively activated. A shift from M2 to M1 has been reported in obesity and inflammation [40]. Increased TNF-*α* upregulates MCP1 expression and leads to adipose tissue macrophage infiltration in obesity [16]. In addition to MCP1, other chemoattractants are also involved in macrophage recruitment to adipose tissues [16]. Some secreted hormones or molecules of adipose tissues are listed in Table 2.

### 3.2. Insulin Resistance in Obesity

**Figure 1:**
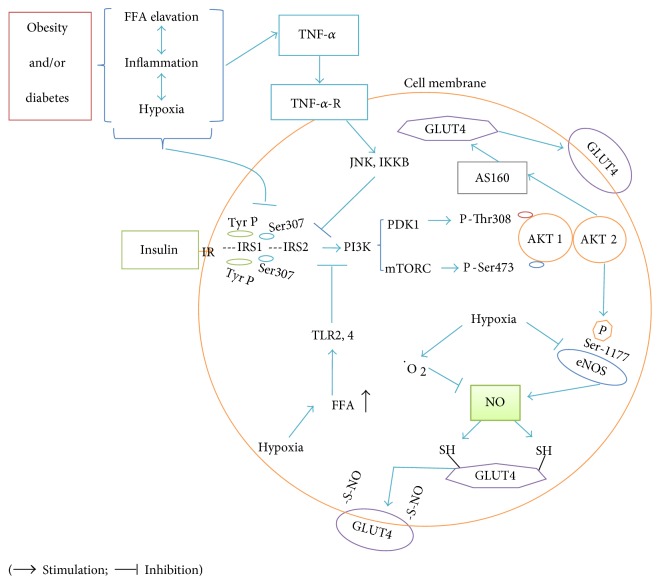
Insulin resistance in obesity. Obesity is associated with hypoxia, inflammation, and lipolysis. These conditions can lead to insulin resistance by impairment of insulin receptor substrate (IRS)/phosphatidyl inositol-3 kinase (PI3K)/AKT pathway. The c-Jun amino-terminal kinase (JNK), Toll-like receptors (TLRs), Akt substrate of 160 kDa (AS160), and AKT/serine (Ser)-1177 are the sensing points that hypoxia and inflammatory factors can inhibit insulin signaling. It should be noted that not all the above signaling occurs in every cell. GLUT: Glucose transporter; IKKB: I*κ*B kinase *β*; IR: Insulin receptor; mTORC: Mammalian target of rapamycin complex; PDK1: 3-Phosphoinositide-dependent protein kinase 1; Ser307: Serine 307; TNF-*α*-R: Tumor necrosis factor-*α* receptor; Tyr P: Phosphorylated tyrosine.

The insulin signaling pathways have been previously reported in detail by several authors [51, 52]. As partly shown in Figure 1, phosphorylation of insulin receptor (IR) tyrosine, protein kinase B (PKB), and Akt substrate of 160 kDa (AS160) in insulin signaling pathways is impaired by hypoxia, changes that are reversible by reoxygenation [53]; hypoxia also inhibits the insulin-induced phosphorylation of IR substrate 1 (IRS-1) and IRS-2 [53]. It has however been shown that the deletion of hepatic prolyl hydroxylase domain enzyme 3 (PHD3) stabilizes the hypoxia-inducible factor-2*α* (HIF-2*α*), a key factor of hypoxia responses, and improves insulin sensitivity [54] (see Stabilization and Destabilization of HIF-1*α*). As shown in Figure 1, hypoxia results in increased inflammatory factors and free fatty acids that lead to insulin resistance via the activation of c-Jun amino-terminal kinase 1 (JNK-1) [55]. The activation of JNK-1 interferes with insulin signaling via phosphorylation of IRS-1 on serine 307 (Ser307) residue [56, 57]. IRS-1 is activated by insulin via tyrosine phosphorylation of IRS-1 in the normal signaling pathway, but the Ser307 phosphorylation of IRS decreases its ability to phosphorylate tyrosine and can therefore cause insulin resistance [57–59]. Hirosumi et al. have reported that Ser307 phosphorylation of IRS-1 is increased in wild type obese mice, but not in *Jnk1^−/−^* mice [57].

Circulating levels of inflammatory factors including free fatty acids and TNF-*α* are higher in obesity [55, 57, 60]; these factors activate JNK-1, resulting in insulin resistance, as aforementioned [59]. It should be noted that TNF-*α* does not directly inhibit IRS-1. The inactivation of JNK-1 in transgenic mice on a high-fat diet leads to increased fatty acid oxidation and energy consumption as well as decreased inflammation [61]. In addition, while basal reactive oxygen species (ROS) level has positive effects on both insulin secretion in *β*-cells and insulin signaling, ROS overproduction is detrimental and can lead to insulin resistance [62–64].

### 3.3. Cellular Stress in Obesity

#### 3.3.1. Mitochondrial Stress

In obesity, the overproduction of ROS leads to adipocyte dysfunction. Increased substrates of the electron transport chain and an increased potential of the mitochondrial inner membrane are the main reasons for increased ROS, in particular the superoxide anion [65]. In addition, high glucose level in some cases of obesity can lead to increased ROS signaling [66]. The superoxide anion is converted to H_2_O_2_ by the enzyme superoxide dismutase; although it is more reactive than H_2_O_2_, the latter can pass across the cell membrane, thereby elevating the ROS levels in cytoplasm and affecting macromolecules [65, 67, 68]. In BAT and brite cells, ROS increases UCP1, which can lower the potential of mitochondrial membrane and can regulate ROS production [65, 69].

#### 3.3.2. Endoplasmic Reticulum Stress

The enlargement of adipocytes leads to increase in protein synthesis, causing endoplasmic reticulum (ER) stress due to inappropriate folding. In addition, chronic high free fatty acid levels can also cause ER stress [70]; ER is a site for the synthesis of proteins, sterols, and lipids; if any of these functions are disrupted, it results in ER stress and can cause apoptosis and *β*-cell death [70, 71], which may negatively affect the insulin production.

## 4. Obese Adipose Tissue Oxygenation

### 4.1. Hypoxic or Hyperoxic Status of the Adipocytes in Obesity

Oxygenation of adipocytes is different depending on their location and types [19]. Ye et al. have reported oxygen pressure (PO_2_) of epididymal fat in lean and obese mice to be 47.9 and 15.2 mm Hg, respectively, a difference indicating ~70% reduction in the latter [72]. Furthermore, it has been reported that hypoxia-induced vascular endothelial growth factor (VEGF) expression is impaired in hyperglycemia/diabetes [73]. A 44% decrease in capillary density and 58% in VEGF mRNA in obese compared to lean individuals indicate that low PO_2_ levels in overweight and obesity do not result in neovascularization [74]. In obesity, free fatty acids are increased [75], which can induce the uncoupling of oxidation from phosphorylation in mitochondrial respiration via induction of uncoupling proteins such as adenine nucleotide translocase 2 (ANT2); ANT2 subsequently increases oxygen consumption, leading to cell hypoxia [76]. There is one report that despite the low blood flow of adipose tissues in obesity, it was suggested that there is an increase in oxygen tension due to low oxygen consumption in mitochondria [77]. Different assay methods (O_2_ electrode versus optochemical measurement) could possibly explain these controversial results. To sum up, several studies have emphasized the hypoxic state of adipose tissues in obesity [4].

### 4.2. Cellular Responses to Hypoxia

Hypoxia can increase cell necrosis and apoptosis in humans and mice. Yin et al. have reported that in vitro hypoxia (1% oxygen for 16 hours) causes 75% cell death in 3T3-L1 adipocytes via increased necrosis (40%) and apoptosis (35%) [34]. In obese mice and humans, adipocyte death is correlated with an increase in adipocyte size and macrophage recruitment [78]. Hypoxia has a key role in initiating obesity disorders through affecting multiple gene expressions (over 1000 genes) in adipocytes [79], in particular HIF-1*α* [76]. Inhibition of ANT2 and/or HIF-1*α* can reverse the complications of obesity, for example, insulin resistance [76]. Furthermore, insulin can induce HIF-1*α* in 3T3-L1 adipocytes by a ROS-dependent mechanism [80]. In addition, ROS (in particular H_2_O_2_) can inhibit PHDs, via oxidation of Fe^2+^ to Fe^3+^, sustaining thereby the activation of HIF-1*α* [81, 82].

#### 4.2.1. Inflammatory Responses to Hypoxia

Hypoxia affects cellular pathways by stimulation of lipolysis, inhibition of adipogenesis, and adipocyte differentiation [19, 83] and consequently increases free fatty acid levels [19]; this issue may be due to the downregulation of PPAR*γ* gene expression in a hypoxic state [83–85]. Furthermore, hypoxia is correlated with an increased expression of macrophage inflammatory protein-1*α* (MIP-1*α*) and macrophage infiltration [74]. In obese mice, hypoxia increases expression of inflammatory genes in M1 macrophages, particularly in the adipose tissues [86]. Hypoxia induces HIF-1*α*-dependent and HIF-1*α*-independent inflammation in visceral but not in subcutaneous fat; this may be due to the presence of M1 macrophages in the former fat and M2 macrophages in the latter fat [86].

#### 4.2.2. HIF-1*α*, a Key Factor for Hypoxia Adaptation in Adipose Tissue

**Figure 2:**
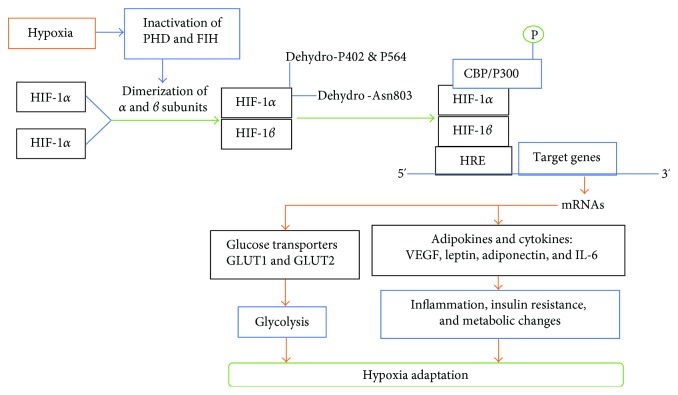
Hypoxia-inducible factor-1*α* (HIF-1*α*) responses to hypoxia. HIF-1*α* acts through up-/downregulation of ~1300 genes including glucose transporters (GLUTs), adipokines, and cytokines. CBP/p300: cAMP response element-binding protein- (CREB-) binding protein (CBP) and p300; Dehydro-Asn803: Dehydroxylated asparagine 803; Dehydro-P402 and P564: Dehydroxylated proline 402 and proline 564; FIH: Factor-inhibiting hypoxia-inducible factor; HRE: Hypoxia-response element; PHD: Prolyl hydroxylase domain enzymes; VEGF: Vascular endothelial growth factor.

*(1) HIF-1α Structure*. HIFs are heterodimers of *α* and *β* subunits [87]. The *α* subunit contains three isoforms, HIF-1*α*, HIF-2*α*, and HIF-3*α*; HIF-1*α* and HIF-2*α* subunits are oxygen sensitive [88]. HIF-1*α* is synthesized in environments with sufficient oxygen levels; however it is targeted for proteasome degradation. The HIF-1*β* subunit is a shared structural and nonoxygen-sensitive subunit that is required for synthesis of the active form of HIF-1*α* [88]. Both *α* and *β* subunits have a Per-ARNT-Sim (PAS) domain, which is involved in HIF-1*α* heterodimerization and DNA binding [87, 88]. The C-terminal side of HIF-1*α* contains two domains for interaction with the cAMP response element-binding protein- (CREB-) binding protein (CBP) and p300 (CBP/p300) [87]. As shown in Figure 2, CBP/p300 recognizes the hypoxia-response element (HRE) in target genes; the products of these genes include cytokines, growth factors, glucose-transporter genes (GLUT1 and GLUT2), and angiogenic genes (VEGF) that are needed for the adaptation to hypoxia [89, 90].

**Figure 3:**
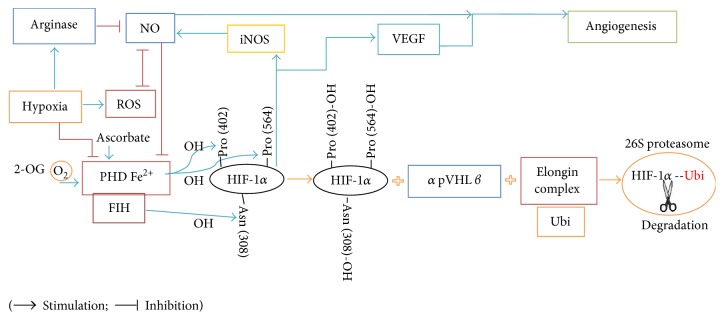
Hypoxia-inducible factor-1*α* (HIF-1*α*) degradation/stabilization. Inhibition of prolyl hydroxylase domain (PHD) enzymes by hypoxia and nitric oxide (NO) leads to stabilization of HIF-1*α*. Hypoxia upregulates the arginase enzyme; thus, the substrate of NO synthase (NOS), arginine, is reduced and NO production is decreased. Furthermore, hypoxia can induce production of ROS (superoxide anion). The bioavailability of ROS and NO is regulated by each other. HIF-1*α* upregulates inducible NOS (iNOS), which produces NO. NO inhibits PHD and stabilizes HIF-1*α*; NO can also contribute to angiogenesis through vascular endothelial growth factor (VEGF), which is upregulated by HIF-1*α*. Asn: Asparagine; Pro: Proline; pVHL: Von Hippel-Lindau tumor suppressor protein.

*(2) Stabilization and Destabilization of HIF-1α*. HIF-1*α* is expressed in all cells, has a half-life of ~1 min, and is regulated via 4-hydroxylation of proline residues (402 or 564) by PHDs, which is oxygen dependent and, in presence of sufficient oxygen, hydroxylates these proline residues [87]. Asparagine-803 residue of the *α* subunit of HIF-1*α* is hydroxylated by the factor-inhibiting hypoxia-inducible factor (FIH) [87]. The 4-hydroxyprolyl HIF-1*α* binds to the *β*-domain of von Hippel-Lindau tumor suppressor protein (pVHL) by a hydrogen bond; then pVHL (from its *α-*domain) binds to Elongin C/B (E3 ligase complex) proteins and induces polyubiquitination of HIF-1*α* [91]. Ubi-HIF-1*α* is considered a signal for degradation in 26S proteasomes (Figure 3) [91]. PHD can hydroxylate the proline residues at several sites of HIF-1*α*, but only some of them (P402 and P564) react with pVHL and cause 26S degradation; interestingly, the full-length HIF-1*α* that contains proline to alanine amino acid sequences, P402A/P564A, is resistant to PHD- and pVHL-mediated degradation [92].

*(3) The Effect of Oxygen and NO on HIF-1α Stabilization*. As shown in Figure 3, the activation of PHD requires oxygen, 2-oxoglutarate (2-OG), and binding to Fe^+2^; this activation is controlled by NO and oxygen levels [91]. In hypoxia, mitochondrial consume oxygen and low oxygen levels inactivate PHD; the hydroxylation of the *α* subunit of HIF-1*α* is therefore attenuated and binds to the *β* subunit to produce the active form of HIF-1*α* [91], which is then translocated to the nucleus and binds to CBP/p300; the complex then binds to HRE and other coactivators and controls the transcription of the genes needed for hypoxia adaptation; decreased FIH and production of dehydroxylated asparagine 803 residue are necessary for the binding of HIF-1*α* and CBP/p300 [87, 91]. In addition, hypoxia stimulates inducible NO synthase (iNOS), and NO overproduction inhibits oxygen consumption by mitochondria; this generates a situation similar to normoxia, where oxygen level is not too low to inactivate the PHD and HIF-1*α* is then degraded in the proteasomes [93, 94]; this mechanism may explain the decreases in HIF-1*α* after the first exposure to hypoxia.

*(4) Other Factors That Influence HIF-1α Stabilization*. The receptor for activated C kinase 1 (RACK1) competes with heat shock protein 90 (HSP90) to bind to HIF-1*α*. RACK1 causes polyubiquitination of HIF-1*α* by binding to Elongin C and ultimately producing Ubi-HIF-1*α*, which is degraded by proteasomes [92]; RACK1-induced degradation of HIF-1*α* is independent of oxygen, pVHL, and PHD. HSP90 competes with RACK1 to bind to residues 81–200 in the PAS-A subdomain of HIF-1*α*; the binding of HSP90 to HIF-1*α* leads to the stabilization of HIF-1*α* by preventing the binding of RACK1 to HIF-1*α* [92].  Table 3 shows the factors that influence HIF-1*α* stabilization.

#### 4.2.3. Reciprocal Influences of HIF-1*α* and Several Cell Pathways and Signals

*(1) The Effects of HIF-1α on the Metabolism of Lipids and Carbohydrates*. HIF-1*α* induces glycolysis (glucose fermentation independent of Krebs cycle) in adipocytes both directly by the activation of enolase and 6-phosphofructokinase and indirectly by the activation of 6-phosphofructokinase, fructose-2, 6-biphosphatase, and aldolase C; HIF-1*α* is also directly involved in the metabolism of lipids (by activation of leptin) and angiogenesis (by induction of VEGF), as well as indirectly disrupting insulin signaling [96]. In HIF-1*α* knockout mice, fed on a high-fat diet, fat mass was decreased, adiponectin induced, and these mice did not develop either obesity or insulin resistance [97].

*(2) The Effect of HIF-1α on BAT and WAT*. HIF-1*α* may have a dual role in the development of obesity-related disorders, because of its different functions in WAT and BAT cells; Zhang et al. reported that increased HIF-1*α* induces thermogenesis in BATs by increasing both VEGF-dependent angiogenesis and mitochondria biogenesis; on the other hand, increased HIF-1*α* increases fibrosis and inflammation in WATs [98]. However, it has been reported that the vascular network is diminished in both BAT and WAT in diet-induced obesity; decreased capillary density is higher in BAT than in WAT, leading to BAT whitening; that is, BAT shows the WAT phenotype [99]. Interestingly, cold induces VEGF expression independent of hypoxia and HIF-1*α* in both WAT and BAT cells by PPAR*γ* coactivator 1-*α* (PGC-1*α*) [100].

Collectively, the overall function of HIF-1*α* is to adjust cell metabolism on low oxygen consumption. The use of hyperoxia or natural nitrate-containing vegetables may be a strategy to reverse obesity disorders; in support of the nitrate intervention, NO has a positive effect on vascular and capillary tone [101] and, unlike BAT whitening factors, causes WAT browning [102, 103].

#### 4.2.4. NO Bioavailability in Hypoxia

NO contributes to vasodilation, vascular remodeling, angiogenesis, and glucose metabolism, as well as playing a protective role in cardiovascular disease [51, 75, 104, 105]. NO is produced by eNOS via the oxygen-dependent pathway, a route which is disabled in a hypoxic state, where NO would be produced via the nitrate-nitrite-NO pathway, to maintain NO bioavailability [106]. Additionally, NO has a very low half-life (~0.05–1.18 ms) [107] and acts locally as an autocrine or paracrine factor, whereas nitrite and nitrate act as endocrine hormones and NO reservoirs [108–110] due to their longer half-lives (110 s and 5–8 h, respectively) [107]. Furthermore, arginine is the common substrate for both arginase and NOS and the increase of arginase activity reduces the arginine needed for NO production by NOS, a status which can cause endothelial dysfunction [105, 111]. Interestingly, arginase is upregulated by hypoxia [111], TNF-*α*, and superoxide anion (derived from uncoupled eNOS) [112], all occurring in obesity and can act together increasing the susceptibility to diabetes in obese individuals. In vitro hypoxia decreases the phosphorylation of serine-1177 and increases the phosphorylation of threonine-495 in eNOS, reducing its activity; furthermore, hypoxia also decreases arginine transporter [111, 113, 114]. In summary, hypoxia decreases NO bioavailability and causes endothelial dysfunction.

#### 4.2.5. NO and Insulin Resistance

Exogenously delivered NO (sodium nitroprusside as a NO donor) stimulates uptake and transendothelial transport (TET) of insulin by inhibition of protein tyrosine phosphatase 1B (PTP1B) via S-nitrosylation; PTP1B dephosphorylates the IRS-1 and IRS-2 tyrosines, as well as inhibiting insulin signaling and TET [115]. Insulin resistance increases mitogen-activated protein kinase (MAPK) activity via blocking phosphatidylinositol 3-kinase (PI3K). PI3K increases eNOS activity and NO production, thereby decreasing insulin resistance; in addition, increased MAPK activity leads to vasoconstriction through endothelin-1 [116]. Thus, insulin resistance can lead to endothelial dysfunction; reciprocally, endothelial dysfunction can cause insulin resistance [117].

Despite previous reports on the carcinogenic effect of dietary nitrate/nitrite [118, 119], no association has been found between nitrate/nitrite and the risk of cancer in some later studies [103, 120, 121]. Inorganic nitrite increases blood flow of pancreatic islets and stimulates insulin secretion [122]. To support the benefits of nitrate intervention, Hezel et al. have demonstrated that long-term dietary nitrate (17 months, NaNO3: 1 mmol/L) improves the response of insulin and concluded that nitrate has no harmful effects on the health of mice [103]. As shown in Figure 1, NO and nitrate can also mimic insulin functions and induce GLUT4 translocation to the cell membrane via nitrosylation of GLUT4 [51].

Elevated free fatty acids in obesity and hypoxia [55] induce inhibitor of kappa B (I*κ*B) kinase *β* (IKK*β*), which phosphorylates serine residues of IRS-1 and interrupts IRS1/PI3k/Akt-dependent eNOS activity that causes NO depletion and impairment of insulin signaling [123]. Free fatty acids also interfere with NO production and insulin signaling by other ways including activating Toll-like receptors (TLRs), in particular TLR4, as shown in Figure 1 [123, 124].

To summarize, obesity and hypoxia cause oxidative stress and NO depletion, leading to endothelial dysfunction, and consequent obesity disorders. Inorganic nitrate/nitrite apart from replenishing decreased NO bioavailability has antioxidant properties; inorganic nitrite reduces superoxide anion bioavailability and iNOS activity [11, 125]. Nitrate administration decreases malondialdehyde (a marker of lipid peroxidation) concentrations and urine concentrations of class VI F2-isoprostanes and 8-hydroxy-2-deoxyguanosine [126, 127]. In addition, nitrite can decrease the formation of vascular ROS, perhaps, by the diversion of electrons away from oxygen [106].

## 5. Advantages and Disadvantages of Hyperoxia Intervention

Normobaric and hyperbaric oxygen therapies (NBOT and HBOT, respectively) have been used in medicine. NBOT has beneficial therapeutic effects on severe acute ischemic stroke [128], and HBOT is therapeutically used in cardiovascular diseases, sleep apnea, wound healing, and management of some tumors [129–131]. Quintero et al. [5] were the first to show that hyperoxia (95% O_2_, 24 h) can increase ROS and proinflammatory adipokines in 3T3-L1 adipocytes; they also showed that hyperoxia upregulates PPAR*γ* that may indirectly have positive effects on insulin sensitivity [5, 9]. Treatment with HBOT (2 atmospheres, 2 h/day, 6 times/week, for 5 weeks) increases insulin sensitivity [132]. NBOT (60% O_2_, 3 days) increases adipocyte survival and regeneration in animal models of fat grafting [133]. Hyperoxia (35% O_2_) can reverse the toxic effects of a high-dose glucose (33.3 mM) on INS1 *β*-cells and restore insulin secretion [134]. Chronic moderate hyperoxia (50% O_2_, for 3 weeks) in male C57BL mice slowed body weight gain and decreased VEGF expression; it however increased HIF-1*α* level during the first week and decreased it after a prolonged exposure [95]. In monosodium glutamate-treated mice, HBOT (2.5 atmospheres, 60 min/d, for 4 weeks with 2 weeks interval) decreased body weight and increased oxidative stress [7]. Furthermore, hyperoxia (36% O_2_ for 3 h daily) in obese type II diabetic rats led to decline in fasting glucose, HbA1c, and the size of adipocytes and increase in metabolic capacity in muscle [135]. A review conducted on the effects of HBOT on traumatic brain injury concluded that HBOT has neuroprotective effects via improvement of tissue oxygenation and cellular metabolism and anti-inflammatory and antiapoptotic properties [136]; hyperoxia (2.4 atmospheres, 90 min) also decreases inflammation in an animal model of inflammatory pain [137]. Hyperoxia depletes 5,6,7,8-tetrahydrobiopterin (BH4) cofactor in the neonatal retina [8]. Bioavailability of BH4 is needed for the normal eNOS function, as BH4 depletion can uncouple eNOS, which produces superoxide anion, thereby decreasing NO production [138]. Some of the other adverse effects of hyperoxia such as inflammation and oxidative stress have been reported [7], as well as anti-inflammatory effects [136, 137]. It is interesting to mention that oxidative stress linked to hyperoxia can have a therapeutic action [139]. Overall, it seems that hyperoxia has important and undeniable advantages, but its safe dose and exposure duration need to be clearly defined.

## 6. Conclusions and Perspectives

Obesity is a chronic hypoxic state, which causes several deleterious changes such as adipose tissue dysfunction, insulin resistance, inflammation, and organ damage, changes which can lead to other metabolic disorders including cardiovascular disease and diabetes. In recent years, to prevent the adverse effects of obesity, some interventions have been suggested, such as drugs, exercise, and healthier diet patterns [140–142]. Although some researchers indicate that high altitudes and hypoxia can lead to weight loss and lower risk of metabolic syndrome [143, 144], these might also have some adverse effects [145] such as decrease in muscle mass instead of fat mass, inflammation, macrophages infiltration, and insulin resistance [146, 147], indicating that not all individuals can adhere to these conditions. Hyperoxia has beneficial effects and can reverse the aforementioned hypoxic status, for example, insulin sensitivity and wound healing [146, 148, 149]. Hyperoxia could be considered as a new strategy for the management of obesity and type 2 diabetes. Exposure to higher doses of oxygen, however, could produce adverse effects [150–152] including eNOS inhibition and increased ROS, both of which could contribute to the development of metabolic disorders and adipocyte dysfunction [153, 154]. Both acute and chronic exposures to hyperoxia have positive effects on carbohydrate metabolism [132, 135], and they can decrease adipocyte size [7, 135]. Nonetheless, there are some differences between the types of exposures; for example, acute exposure has anti-inflammatory effects, while chronic exposure has the opposite effects [5, 9, 137]. Furthermore, the effects of hyperoxia on oxidative stress and VEGF have been reported in the chronic treatment [7, 95]. In addition, there is a report suggesting that hyperoxia during the first week of treatment increased HIF-1*α* and HIF-2*α* levels, which were restored to near normal values at 2 to 3 weeks of exposure [95]. It has also been shown that hyperoxia inhibits HIF-1*α* protein expression and DNA-binding activity, in rat INS-1 *β* cells [134]. Further studies are needed to elucidate the acute and chronic effects of hyperoxia.

Recent data support the beneficial effects of a nutritional-based nitrate/nitrite therapy in obesity and diabetes [108, 155]. Nitrate has also antioxidant properties [11], and it can restore NO depletion induced by hyperoxia. Therefore, for the management of obesity, it seems reasonable to combine hyperoxia and nitrate. To the best of our knowledge, there is no study to address the effect of this combination therapy to manage obesity/diabetes, warranting the need for evaluating the effects of hyperoxia with different durations and oxygen pressures [7, 156, 157], viz. on hyperbaric versus normobaric hyperoxia, simultaneously with nitrate intervention.

## Figures and Tables

**Table 1:** Some characteristics of the three types of adipocytes.

	White adipocytes	Brown adipocytes	Brite or beige adipocytes	References
Origin in adulthood	Mesenchymal and endothelial precursors	Mesenchymal precursors, muscle satellite cell, and endothelial precursors	WAT adipocyte and endothelial precursors	[12, 14]
Transcription factor	Myf5^−^ and Tcf21	Myf5^+^	Tcf21	[14]
Specific gene expression	Leptin	UCP1 and Zic1	UCP1 and Hoxc9	[12, 20, 21]
Number of mitochondria	Low	High	High	[12, 13]
Main function	Lipid storage	Heat producer	Heat producer	[12, 13]
Effect on obesity	Obesogenic	Antiobesity	Antiobesity	[13]
Histological phenotype	Large cells with one huge lipid vacuole	Small cells with several lipid vacuoles	Small cells with several lipid vacuoles	[14, 20]
Anatomical description of fat depots (mice)	Epididymal, mesenteric, inguinal, retroperitoneal, and cardiac	Interscapular, axillary, cervical, and mediastinic	Inguinal, cardiac, and retroperitoneal	[21]

Hoxc9: homeobox9; Myf5: myogenic factor 5; Tcf21: transcription factor 21; UCP 1: uncoupling protein 1; WAT: white adipose tissue; Zic1: zinc finger protein in the cerebellum 1.

**Table 2:** Some adipose tissue secreted adipokines or cytokines.

Adipo/cytokines	Function	References
Adiponectin	Increases *β*-oxidation, insulin sensitivity via AMPK, increases glucose uptake, and glucose tolerance. Decreased adiponectin is related to obesity, TNF-*α* upregulation, and eNOS downregulation.	[41, 42]
Sfrp5	Is increased by calorie restriction diet and has an anti-inflammatory action.	[43]
Adipolin	Is known as adipose-derived insulin-sensitizing factor, improves glucose metabolism, and decreases insulin resistance and inflammation.	[44]
Apelin	Inhibits diet-induced obesity, due to its improvement of vascular integrity. It is positively correlated with BMI, and it is upregulated by insulin in obesity.	[45–47]
PPAR*γ*	Induces storage of lipids and adipogenesis and reduces lipotoxicity; it also regulates whole body insulin sensitivity.	[48]
Leptin	Is a cytokine-like hormone, which inhibits food intake and energy expenditure. It impairs NO-mediated component.	[49]
Resistin	Is increased in genetic- and diet-induced obesity models. It is specific for WAT and causes insulin resistance.	[50]

AMPK: adenosine monophosphate-activated protein kinase; BMI: body mass index; eNOS: endothelial nitric oxide (NO) synthase; Sfrp5: soluble (secreted) frizzled-related protein 5; WAT: white adipose tissue.

**Table 3:** Factors influencing HIF-1*α* stabilization.

Effectors	HIF-1*α* stabilizer	HIF-1*α* destabilizer	References
Oxygen		✓	[87]
2-OG (2-oxoglutarate)		✓	[87]
Hypoxia	✓		[87]
Hyperoxia		✓^∗^	[95]
iNOS-derived NO		✓	[94]
RACK1		✓	[92]
HSP90	✓		[92]
ROS	✓		[81, 82]

HIF-1*α*: Hypoxia-inducible factor-1*α*; HSP90: Heat shock protein 90; iNOS: Inducible nitric oxide synthase; RACK1: Receptor for activated C kinase 1; ROS: Reactive oxygen species.

^∗^HIF-1*α* gene expression increases in the first week of hyperoxia exposure and restores to near normal values in prolonged hyperoxia exposure in weeks 2-3.
